# Perceived caregiver’s burden among children with autism spectrum disorder in central Nepal: a cross-sectional study

**DOI:** 10.1097/MS9.0000000000000603

**Published:** 2023-04-11

**Authors:** Manju Shrestha, Navina Shrestha, Yugant Khand, Lhamu Sherpa

**Affiliations:** a Center for Autism, Baluwatar; b All Nepal Football Association, Satdobato; c Nepalese Army Institute of Health Sciences, College of Medicine, Sanobharyang, Kathmandu, Nepal

**Keywords:** autism spectrum disorder (ASD), burden, caregivers

## Abstract

Autism spectrum disorder (ASD) is the most common childhood neurodevelopmental disorder characterized by an atypical social and communicative functioning and restricted, repetitive patterns of behaviour activities. Caring for children with ASD is challenging for both parents and their caregivers. This study aims to explore the psychosocial burden of the caregivers of children with ASD.

**Materials and methodology:**

An analytical cross-sectional study was performed in Centre for Autism in Kathmandu, Nepal. The enrolment occurred between January 2022 and July 2022 among the caregivers of children with ASD. One hundred twenty caregivers in contact with the centre was evaluated using the Zarit Burden Interview-22 during the study period meeting inclusion criteria.

**Results:**

Our study showed that majority of caregivers among child with ASD were mothers 65 (54.16%, *n*=65) followed by grandparents 35 (29.16%, *n*=35) and father 13 (10.8%), respectively. Among them, most of the caregivers perceived moderate to severe burden 57 (47.5%) followed by mild to moderate burden 45 (37.5%) and only 7 (5.8%) of the caregivers perceived severe burden during the study which was found to be statistically significant.

**Conclusions:**

This study highlighted the fact that although most of the caregivers perceived moderate to severe burden while caring a child with ASD. The degree of burden significantly correlated with the level of ASD in the child.

## Introduction

Highlights

- Autism spectrum disorder (ASD) is a chronic condition requiring comprehensive care from clinicians and families.
- Moderate level of ASD showed highest level of parental moderate to severe burden.
- Majority of children belonged to moderate followed by high and low level of ASD.
- Mothers were the major caregivers followed by grandparents and fathers.
- Caregivers perceived mild to moderate burden while caring a child with ASD.
- Level of burden significantly correlated with the level of ASD in the child.

ASD is a prevalent neurodevelopmental disorder with core deficits identified in social communication or interaction and restrictive, repetitive patterns of behaviour^1^. The increasing prevalence of ASD has been the subject of interest in the medical literature and lay press^2^. ASD can be diagnosed as early as 14–16 months. Prompt diagnosis with early interventions has proven to show promising results^3^. There are several patterns where parenting a child with ASD differs from parenting a child without ASD. Considering the course of autism from childhood to adulthood, they require long-term medical care with the help of specialized primary care physicians. Although caregiving is an essential element of parenting, there is a physical and mental impact on the families. Certain functional limitations may necessitate structural or technical modifications in the physical home environment^4^,^5^.

With ASD becoming prevalent in recent decades, it is essential to understand the facade that caregivers bear^5^. The management of ASD lacks unified and guided approach for its treatment in Nepal. This is partly due to the diverse spectrum of presentation in different age group, sociocultural influence, and failure to implement policies that support the families of children with ASD. A child with ASD has difficulty in their ability to learn and integrate; consequently, they rely on parental care for extended periods of their lifetime^6^.

The burden of living with an autistic child may produce a state of permanent hurt, shock and grief for the caregivers. The families are often predisposed to public embarrassment^7^. As the primary caregiver of children with ASD, mothers usually face a spectrum of problems like a heavy financial burden, discrimination, stigma and ineffective therapy^8^–^11^. Their lives become severely limited with consequent stress on their marriage, which affects the mental health of the families^4^,^5^. A foreboding financial crunch also affects the nature of caregiving that a family could otherwise afford when working in capacity. The distress caused by caregiving, deteriorating quality of life, adverse effects on professional and social life and psychological stress are the burdens faced by the caregivers^12^.

This study aims to explore the overall psychosocial burden among caregivers of children with ASD studied in central Nepal.

## Material and methodology

### Study design and setting

We performed an analytical cross-sectional study in the Centre For Autism (CFA) from January 2022 to July 2022. CFA is an autism centre founded in 2019 in Kathmandu, Nepal that manages children diagnosed with ASD. CFA is the first inclusive private centre of its kind in Nepal where children can study while undergoing therapy for ASD. The facility provided by the centre includes, special school for children with ASD, multiple therapies like occupational therapy, sensory integration therapy, speech therapy, and behavioural therapy. There are a total of 87 children with autism in school and around 25–30 autistic children come daily for different therapies mention above. The permission for the study was obtained from the Institutional Review Board of CFA.

### Study population and sampling

A total of 135 children diagnosed with ASD were enroled in our study with the help of the centre. The details were obtained from the family caregivers of the selected children. The enrolment occurred between January 2022 to July 2022.

The caregivers of children above 18 months of age were provided with a questionnaire (*N*=120). Those respondents who incompleted the questionnaire, caregivers of children with ASD under the age of 18 months and caregivers without consent were excluded from the study (*N*=15).

### Measurement

The proforma consisted of three subheadings. The first part included an inquiry regarding general information and sociodemographic status. The second part included anxiety and depressive symptoms of the mother and caregiver using Zarit Burden Interview-22 (ZBI-22). The ZBI-22 technique is composed of 22 items that reflect how caregivers feel when taking care of their children. The ZBI-22 was modified in a certain context of reference from “your relative” to “your child” in all the 22 statements. The scale includes five categories of responses (never, rarely, sometimes, quite frequently, nearly always, rated 0–4, respectively). The total score ranges from 0 to 88. A total score was calculated by the sum of all the item scores and a high score indicates a higher level of burden. Scores were interpreted as 0–20 for no burden, 21–40 for mild to moderate burden, 41–60 for moderate to severe burden and 61–88 for severe burden. Table 1

**Table 1:** ZBI cutoff scores and their interpretation.

Caregiver’s burden	Interpretation
0–20 score	No burden
21–40 score	Mild to moderate burden
41–60 score	Moderate to severe burden
61–88 score	Severe burden

ZBI, Zarit Burden Interview.

Lastly, the core symptoms of children with ASD are evaluated using Autism Diagnostic Observation Schedule-Second Edition. Autism Diagnostic Observation Schedule-Second Edition is a standardized assessment tool that helps providers diagnose and determine the severity of ASD in children. Table 2.

**Table 2:** Level of ASD based on ADOS-2

Level of ASD	Score
Mild autism symptom	1–4
Moderate autism symptom	5–7
High autism symptom	8–10

ADOS-2, Autism Diagnostic Observation Schedule-Second Edition; ASD, autism spectrum disorder.

Our study is reported in line with the STROCSS criteria^13^.

## Data analysis

The analysis of the data were performed in two phases. First, for demographic profile of the caregivers and the children with ASD. In the second phase, logistic regression analysis was used to assess relationship between severity of autism with ZBI-22. Relationships were considered significant if *P* is less than 0.05. All analyses were performed using the Statistical Package for the Social Sciences SPSS v 20.0.

## Results

A total of 120 participants were included in the study. Our study showed that majority of caregivers among child with ASD were mother 65 (54.16%) followed by grandparents 35 (29.16%) and father 13 (10.8%), respectively. Majority of caregivers belonged to age group between 31 and 40 years (42.50%). The mean age of the caregivers was 39 ± 4.7 years (range 19–73 years). 32 (26.66%) participants received higher education and 13 (10.86%) had attained university degree. 10 (8.33%) and 30 (25%) participants were employed in either governmental or private jobs respectively while 65 (54.16%) caregivers were housewife. This study also showed that most of the caregivers belonged to urban sector 97 (80.83%). This study also highlighted the higher socioeconomic status among the caregivers. Table 3.

**Table 3:** Sociodemographic status of the caregivers of ASD children

Variables	*N*	Percentage (%)
Type of caregivers		
Father	13	10.8
Mother	65	54.16
Grandparents	35	29.16
Relatives	7	5.8
Age in years		
<20 years	7	5.8
21–30 years	35	29.16
31–40 years	51	42.50
>40 years	27	22.5
Education		
Illiterate	5	4.16
Primary	25	20.83
Secondary	45	37.5
Higher	32	26.66
University or degree	13	10.83
Occupation of caregivers		
Housewife	65	54.16
Government job	10	8.33
Private job	30	25.0
Others	15	12.5
Economic status		
Upper	15	12.5
Middle upper	65	54.16
Lower upper lower	25	20.83
Lower middle	10	8.33
Lower	5	4.16
Marital status		
Married	93	77.5
Widow	4	3.33
Divorced	23	19.16
Residence		
Urban	97	80.83
Rural	23	19.16

ASD, autism spectrum disorder.

Our study highlighted that among 120 children diagnosed with ASD, majority of children belonged to moderate level of ASD 73 (60.83%) followed by high 32 (26.66%) and low 15 (12.51%), respectively. Table 4.

**Table 4:** Level of ASD based on ADOS

Level of ASD	*N* (%)
Mild autism symptoms	15 (12.51)
Moderate autism symptoms	73 (60.83)
High autism symptoms	32 (26.66)

ADOS, Autism Diagnostic Observation Schedule; ASD, autism spectrum disorder.

According to the evaluation of burden in caregivers, most of them perceived moderate to severe burden 57 (47.5%) followed by mild to moderate burden 45 (37.5%) Table 5 Only 7 (5.8%) of the caregivers perceived severe burden during the study.

**Table 5:** Interpretation of caregiver’s burden

Caregiver’s burden	*N* (%)
0–20 score	11 (9.1)
21–40 score	45 (37.5)
41–60 score	57 (47.5)
61–88 score	7 (5.8)

Likewise, our study showed that there is a significant relation (*p* = 0.021) between caregiver’s burden with child’s level of ASD. The study showed higher level of burden among children diagnosed with high level of ASD Table 6.

**Table 6:** Relationship of Caregiver’s burden with Level of ASD

Level of ASD	No burden	Mild to Moderate	Moderate to Severe	Severe	Total	*P*
Low	10 (90.90)	5 (11.11)	—	—	15 (12.5)	
Moderate	1 (9.09)	39 (86.66)	31 (54.38)	2 (28.57)	73 (60.83)	0.021
High	—	1 (2.22)	26 (45.61)	5 (71.42)	32 (26.66)	
Total	11 (9.16)	45 (37.5)	57 (47.50)	7 (5.8)	120 (100)	

ASD, autism spectrum disorder.

## Discussion

The definite diagnosis of ASD is made clinically by a specialist who meet the Autism Diagnostic Observation Schedule criteria based on history and observation of behaviour. ASD is a chronic condition requiring comprehensive management with hauling efforts from both clinicians and family members or caretakers. The management has no definite principles but ASD specialists cater treatment according to the child’s needs. A common analogy with previous literature, this study showed that a significant caregivers showed moderate to severe burden in caring for their child with ASD. With personal, professional and familial affairs on a daily basis, the burden of raising a child with ASD comes with challenges. A study highlights that a demanding effort is required from the caregiver because a significant prevalence is seen among the siblings of ASD ranging up to 20%^14^–^16^.

In our study, 54% mothers were the major caregivers for a child with ASD followed by 29% being grandparents and 10% were fathers. This creates a gap in the literature where the burden between mothers and fathers cannot be wholly generalized as there are differing domestic roles of mothers and fathers in Nepal which can vary the level of burdens each face. Another study also corroborates that females were the majority caregivers in children with ASD^17^. The caretakers of ASD dedicate a wholesome time for their children and require some measure of assistance from their parents and other family members^18^. In our study, grandparents played a significant role as caregivers than fathers. The quality of life of caregivers are also impacted by the overwhelming nature and duration of the disease. With regards to parental care, mothers of children with ASD have shown increased level of involvement in the care of the child and showed higher levels of mental stress as compared to the fathers. Our study showed mothers followed by grandparents and fathers to be the major caregivers. In Nepal, mothers and grandparents have a significant role in the upbringing of a child while the father is usually looking after the finances for the family.

In comparison to mothers of normally developing children, mothers of ASD devote a significant time in providing care and completing household work and also participate less time in leisurable activities^19^,^20^. Mothers experience higher anxiety levels and depression than fathers^21^. Another study suggested that mothers were more in clinical range for depression than fathers with ASD^22^.

Our study demonstrated that nearly half of the caretakers were graduate of higher education or had attained university degree. The caretakers had a considerable source of income from their private or governmental jobs while most mothers were homemakers. A similar study conducted in Nepal showed that employment has no significant association with the caregiver’s burden^23^.

Majority of the diagnosed children were from the urban area with a higher socioeconomic status. A study conducted in Nepal showed that caregivers aged over 25 years with secondary education level or higher has more knowledge about the nature of autism^24^. The family members anchor on the management laid by the specialist but have a challenging course that toll on their personal and professional careers.

A study also indicates that healthcare expenditure in children with ASD are relatively higher than for individuals with other mental health condition^25^.

A number of reasons can contribute to the overall cost such as loss of productivity in caregiver with subsequent loss of income thereafter. However, each family member is affected in multitude of financial and personal domains. Caregiver burden is another determinant in developmental outcome of child with ASD. Our study highlighted that among 120 children diagnosed with ASD, majority of children belonged to moderate level of ASD 73 (60.83%) followed by high (26.66%) and low (12.51%), respectively.

We concluded that 47% of caregivers felt moderately to severely burdened and 37% experienced mildly to moderately burdened while 9% felt no burden in looking after their child with ASD. Researchers have found that caregivers often respond with coping strategies to stressors while parenting a child with ASD. These can be grouped into problem-based coping and emotion-based coping mechanism^26^. The use of emotion-based coping methods like denial were found to be associated with higher levels of psychological distress while problem-based coping methods like planning and stratifying to counter the problems led to improved health outcomes. The overall burden was mainly seen in higher levels of ASD. Our study showed than an insignificant portion of caregivers were severely burdened and this is mainly attributed to the social dogma in Nepal which involves interacting and offering support from other family members in the care of a child. A direct predictor of burden is the severity of autistic symptoms. It can be reflected from our study that moderate level of ASD showed highest level of parental moderate to severe burden. Our finding corroborates the view that as severity in ASD increases, the level of burden also increases. However, discrepancies due to inadequate variation in the sample and differences in the age group of children with ASD can potentially affect the result.

The burden among parents and families reciprocally and negatively impacts the diagnosed child and can even serve to diminish the positive effects of intervention. However, most interventions for ASD are evaluated only in terms of child outcomes, ignoring parent and family factors that may have an influence on both the immediate and long-term effects of therapy. Therefore, recent management aims at targeting child outcomes along with parent or family outcomes. This newly introduced domain targets family functioning, parent child relationship, preventing stressors, parenting efficacy and parental mental health. The condition in itself is a pandora of unparallel challenges that the parents and healthcare providers address like no other condition.

Regardless of the fallbacks in the study, the study highlights that caregivers can experience a spectrum of burden from mild to severe in taking care of a child with ASD and primary physicians or specialists should consider parental or caregiver’s factor while setting the management parameters.

## Strengths and limitation

This study included participants from diverse backgrounds and family caregivers were in in touch with CFA on regular basis. CFA being a first kind of inclusive school for children with ASD and clinical management within the premise, this helped us reach maximum number of families for the study.

However, there are few limitations in this study. Firstly, the sample population reflects a minor proportion of children with ASD and it does not truly reflect the children with ASD from rural areas of Nepal. Secondly, all measurements were self-reported by caregivers which predisposes to response bias. Lastly, the duration since diagnosis of ASD was not taken into consideration which could have a significant impact on the level of burden perceived by the caregivers.

## Conclusions

Our study highlighted the fact that though most of the caregivers’ perceived moderate to severe burden while caring a child with ASD, the level of burden significantly correlated with the level of ASD in the child. Due to the varied challenges faced by family members in caring for a child with ASD, certain policies directing.

## Ethical approval

This study was approved by the Institutional Review Board from Center For Autism, Kathmandu, Nepal.

## Consent

Written informed consent was obtained from the patient for publication of this case report and accompanying images. A copy of the written consent is available for review by the Editor-in-Chief of this journal on request.

## Source of funding

This study received no funding of any kind.

## Author contribution

M.S.: conceptualization, supervision. Y.K.: writing—original draft. All auithors: Writing—review and editing. All the authors read and approved the final manuscript.

## Conflicts of interest disclosure

The authors declare that there are no conflicts of interest in this study.

## Research Registry Unique Identifying number (UIN)

1. Name of the registry: Research Registry (http://www.researchregistry.com).
2. Unique Identifying number or registration ID: researchregistry8485.
3. Hyperlink to your specific registration: https://researchregistry.knack.com/research-registry#user-researchregistry/registerresearchdetails/63706c1499536d0024ba025d/

## Guarantor

Manju Shrestha.

## Provenance and peer review

Not commissioned, externally peer-reviewed.
